# Fitness Tracker Information and Privacy Management: Empirical Study

**DOI:** 10.2196/23059

**Published:** 2021-11-16

**Authors:** Mohamed Abdelhamid

**Affiliations:** 1 Department of Information Systems California State University, Long Beach Long Beach, CA United States

**Keywords:** privacy, information sharing, fitness trackers, wearable devices

## Abstract

**Background:**

Fitness trackers allow users to collect, manage, track, and monitor fitness-related activities, such as distance walked, calorie intake, sleep quality, and heart rate. Fitness trackers have become increasingly popular in the past decade. One in five Americans use a device or an app to track their fitness-related activities. These devices generate massive and important data that could help physicians make better assessments of their patients’ health if shared with health providers. This ultimately could lead to better health outcomes and perhaps even lower costs for patients. However, sharing personal fitness information with health care providers has drawbacks, mainly related to the risk of privacy loss and information misuse.

**Objective:**

This study investigates the influence of granting users granular privacy control on their willingness to share fitness information.

**Methods:**

The study used 270 valid responses collected from Mtrurkers through Amazon Mechanical Turk (MTurk). Participants were randomly assigned to one of two groups. The conceptual model was tested using structural equation modeling (SEM). The dependent variable was the intention to share fitness information. The independent variables were perceived risk, perceived benefits, and trust in the system.

**Results:**

SEM explained about 60% of the variance in the dependent variable. Three of the four hypotheses were supported. Perceived risk and trust in the system had a significant relationship with the dependent variable, while trust in the system was not significant.

**Conclusions:**

The findings show that people are willing to share their fitness information if they have granular privacy control. This study has practical and theoretical implications. It integrates communication privacy management (CPM) theory with the privacy calculus model.

## Introduction

### Background

Fitness trackers are wearable devices that allow people to monitor and track activities and information related to fitness, such as distance walked and calories consumed. Fitness trackers can be stand-alone devices or integrated within a smartwatch. The device is usually connected to a mobile app that allows users to manage information and use the features of the app. The first functional fitness tracker was invented in the mid-1960s [1]. However, the rise of fitness trackers and wearable devices started about a decade ago. The first Fitbit was released in 2012 [2], and the first Apple watch was revealed in 2014 [3]. Since then, fitness trackers and wearable devices have become increasingly popular. A recent Pew Research Center study reported that 21% of Americans regularly use fitness trackers or smartwatches [4]. Similarly, in 2019, Gallup reported that more than one in four Americans use an app or device to track fitness-related activities [5].

People use fitness trackers for various reasons, but ultimately, the main reason is to get fit or maintain health [6,7]. The device/app helps users stay motivated and allows them to track progress and stay informed [8]. In recent years, many people have started seeking a healthier lifestyle and adopted technologies that motivated them to keep track of their goals [9]. This trend is largely adopted by millennials; some even called the millennials “the wellness generation” [10].

Information collected by fitness trackers and maintained by their respective systems, if shared with health care providers, could provide wider benefits to users. Sharing personal fitness data with health care providers allows physicians to better understand their patients’ health lifestyle, health issues, and potential health problems. This further allows physicians to provide early recommendations and make better health assessments that could help people avoid health problems. In general, sharing personal fitness information with health care institutions may benefit individuals in many ways, such as better health outcomes and reduced cost. Researchers are starting to predict a future that encourages patients to share fitness data with their providers [11]. Another possible benefit of sharing fitness data is to conduct scientific research that could improve the health outcomes for the general public.

However, the use of technology and the sharing of personal fitness data may result in negative consequences, mainly related to privacy and security [12]. For example, loss of privacy may result from a breach of security in a health care institution. More than 93% of health care institutions have been victims of a data breach in the past 5 years [13].

This study investigates the influence of implementing granular privacy control on users’ intention to share their fitness information and whether it could lead to higher user engagement in sharing fitness data. In addition, the paper investigates the motivation of individuals, captured by the privacy calculus, to share fitness information. This research provides several important contributions to the field. First, it contributes to theory by integrating communication privacy management (CPM) theory with the privacy calculus model [14]. Second, the paper provides practical and theoretical insights into how to address barriers to fitness information sharing by suggesting flexible sharing mechanisms that mitigate the impact of perceived privacy risk.

### Theoretical Background and Hypothesis Development

#### Privacy in eHealth

The diffusion of health-tracking apps and devices is growing rapidly in the United States, both in terms of the number of people that are active end users and also in terms of the functionalities and features of the apps and devices that are currently available. Fitness and health-tracking apps are expected to have many benefits for the users, such as motivation and fitness aspiration [15]. However, with digitization come the risks of privacy and security breaches [16,17]. People’s behavior with regard to sharing personal health information is negatively influenced by concerns over their privacy [18,19]. Individual-centered privacy research found that individuals are concerned about the collection, handling, and possible unauthorized access of their private information [18].

Research on health information sharing summarizes that risks of privacy invasion and information violations are drawbacks of sharing [20]. This means that individuals are hesitant to share personal health information because of possible privacy risks. Although sharing is beneficial in many cases, the risks may outweigh the benefits, and thus risk can drive adoption [18]. Therefore, granular privacy control mechanisms for information sharing may motivate individuals to share more of their fitness information. Cavusoglu et al [21] found that in the social media context, granular control motivates users to share more information because they control with whom they share information. This research covers the gap by testing for the impact of a more granular privacy control in sharing fitness tracker information.

#### Information Sharing

One body of literature focused on the sharing of health-related information via online and electronic sources. Prior research suggests that privacy concerns are the central obstacle to sharing of information [18,22,23]. Simon et al [24] identified privacy and security issues and lack of benefits as the main barriers to sharing of personal information.

Angst and Agarwal [18] confirmed that privacy concerns reduce the likelihood of sharing health information. Weitzman et al [25] found that patients with sensitive information are less likely to share their health information with health care providers. Likewise, Zulman et al [26] confirmed that willingness to share health information is influenced by the type of information. Bansal and Gefen [23] reported that the sensitivity of information influences individuals’ decisions to share that online.

The key factors in sharing health information include the benefits of obtaining feedback related to potential health issues. According to Dimitropoulos et al [27], most people realize the benefits of sharing health information. However, they need to adapt to and manage the way their information is shared. Although Ancker et al [28] found that most respondents believe that sharing health information improves the quality of care.

#### Privacy Calculus Model

Information sharing and privacy have always been supplementary [29,30]. Therefore, the theoretical model of this research is guided by the privacy calculus model [14]. Studies in various contexts have used the privacy calculus model [31]. For example, Kim et al [32] used the privacy calculus model to investigate people’s willingness to provide personal information in the context of the internet of things (IoT). They found that perceived benefits are a strong motivator in sharing private information. Likewise, Fox [33] investigated the influence of privacy calculus variables on individuals’ intention to adopt mobile health technologies. The paper finds a stronger influence of benefits compared to risks and concerns. Abdelhamid et al [34], when examining factors associated with the sharing of health information, found that privacy is the biggest barrier to sharing.

The privacy calculus model is a good fit for this study because it deals with information sharing in scenarios where risk and benefits of sharing are involved. However, the privacy calculus model does not incorporate granular control of information into the model. Thus, this research integrates CPM, which incorporates control of information, with privacy calculus, which deals with risks and benefits. Therefore, the overall model covers the three key factors of this research: granular control, risks, and benefits.

This research adopts the theory by:

1. Applying the theory in the context of fitness trackers and wearable devices
2. Integrating CPM theory with the privacy calculus model

#### CPM

CPM theory describes the rationale behind an individual’s choice to disclose or withhold private information [35].

The principles state that people believe they are the ultimate owners of their private information and that people have the right to control the course of their private information. Thus, people believe they have the right to choose with whom to share information. CPM also argues that people believe that stakeholders accessing an individual’s shared information will follow current and future privacy rules. In addition, CPM debates that violated privacy rules will result in negative consequences, including mistrust and uncertainty. Petronio [35] underlined that CPM was developed to help users make necessary alterations to systems when faced with the issue of privacy.

**Figure 1:**
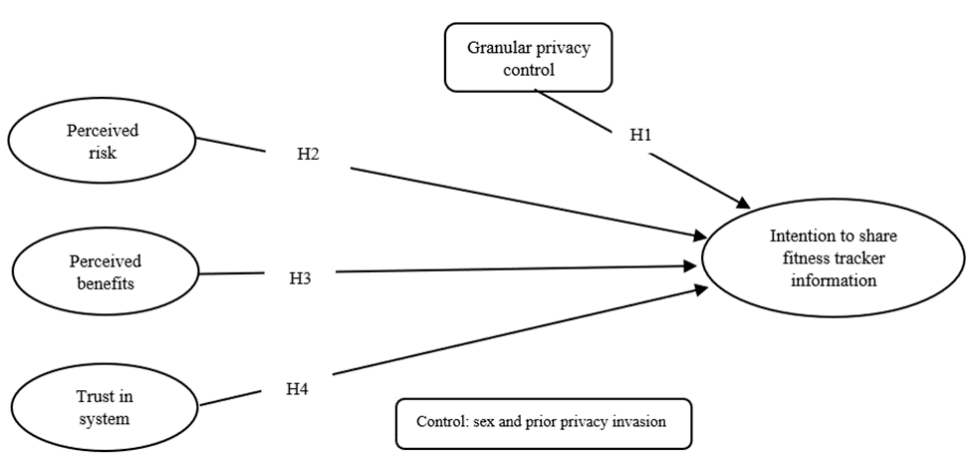
Conceptual model. H: Hypothesis.

According to the breach data reported to the Department of Health Human Services, the number of unauthorized access/disclosure breaches tripled in 2017 versus 2012 [36]. Dhopeshwarkar et al [37] noted that most people want to know who is viewing their information. Angst et al [38] suggested that institutional factors and IT investments affect the likelihood of breaches in health care organizations. This research investigates the impact of a more granular privacy control on the intention to share data related to fitness activities with health care providers. Figure 1 shows the conceptual model of this study.

#### Privacy Control

When faced with a choice, individuals typically choose the option with the premier value after assessing likely risks [39]. In the context of fitness and health and from an individual perspective, there may be a desire to protect specific fitness information from certain providers or users. Caine and Hanania [40] suggested that people prefer to share particular health-related information with specific recipients.

The definition of privacy refers to the right that people have in choosing the information they want to share and with whom to share it [41]. Information control and information disclosure have a positive relationship in various contexts [42,43]. For instance, Cavusoglu et al [21] examined the causal effect of granting Facebook users more control on information-sharing behavior. They found that privacy control increases the open release of information. In other words, when the sharing decision is universal, people might not share some information with anyone. That is mainly because some people want to prevent others from seeing that information. As a result, the decision led to withholding the information from everyone. However, when individuals are given more control over their information, they will share some information with some people.

In addition, Slovic [44] found that on average, individuals are ready to take more risks when they are in control. The paper suggested that improved control increases an individual’s willingness to participate in that behavior. Likewise, Brandimarte et al [45] reported that individuals are more willing to reveal sensitive information when they have more control over what is being shared.

The first hypothesis of this study is as follows:

*Hypothesis 1:* Granular privacy control will yield a higher intention to share fitness information with health care providers.

#### Perceived Risk

The concept of perceived risk has been studied in many contexts in which individuals may face a risky decision. A considerable number of studies have established the link between perceived risk and information disclosure, in general [46-48]. Dinev and Hart [14] defined perceived risk as the perceived risk of unprincipled behavior related to the sharing of personal information. In the context of fitness tracker information, the risk includes selling the information to a third party, misuse, and unauthorized sharing. In 2015, the National Telecommunications and Information Assurance (NTIA) surveyed approximately 40,000 participants [49]. The report stated that more than 50% of users had limited their online activities due to concerns about the privacy of their information.

The second hypothesis of this study is as follows:

*Hypothesis 2:* Perceived risk will have a negative influence on the intention to share fitness information with health care providers.

#### Perceived Benefits

When people face a decision that involves sharing of private fitness information, they usually assess the risks and benefits of sharing to make an informed decision. Many benefits result from sharing fitness information with health care providers. Some of those benefits directly influence the individuals sharing the fitness information. For example, fitness information can help doctors make better health assessments with regard to the person sharing the information. This allows doctors to make better recommendations. The individual may then benefit from better health outcomes, in general.

In the context of fitness, perceived benefit is defined as the perceived value that individuals attach to sharing personal fitness information with health care providers. In general, perceived benefits have been associated with information sharing in various contexts, including health care [50]. For example, Wang et al [51] reported that patients find that improved health care quality and convenience are among the benefits of sharing personal health information. Likewise, Zhang et al [52] found that sharing health information in online communities is associated with benefits for users. They argue that in online communities, the benefits are informational and emotional support. Morris et al [53] proposed a design of a mobile information-sharing system for emergency rooms. They found that sharing can be beneficial for physicians in terms of reducing information-seeking time and stress. This could result in better care for patients. As a result of sharing, patients may be able to avoid a serious problem.

The third hypothesis of this study is as follows:

*Hypothesis 3:* Perceived benefits will have a positive influence on the intention to share fitness information with health care providers.

#### Trust in a System

Trust in a system is defined as the extent to which individuals are confident that systems will handle their information securely and reliably [14]. The prior literature has established a positive relationship between trust in a system and engagement with the system [54,55]. The perception of trust can be linked with the system itself or with the system’s capability to protect information from people who breach the system to misuse information. The perspective of information misuse escalates when private fitness information is exchanged from one system to another. Gefen et al [56] state that the relationship between trust in a system and the intention to use that system becomes more significant when engagement includes the possibility of risk consequences.

The fourth hypothesis of this study is as follows:

*Hypothesis 4*: Trust in the system will have a positive influence on the intention to share fitness information with health care providers.

## Methods

### Data Collection

This study uses scenario-based survey data collection through Amazon Mechanical Turk (MTurk). A survey-based approach has been used in many studies in the context of health care IT to understand individuals’ perceptions related to information sharing [18,20]. This study aims to understand individuals’ perception and intentions as they relate to sharing fitness tracker information. Thus, a survey-based study is adequate. Many studies in the health care field have used Amazon MTurk to collect data [57]. Online data collection is relevant to this study for many reasons. First, fitness trackers are used by the general public and not restricted to a certain occupation or demographic. Second, most people in the United States have regular access to the internet [58]. Third, online data collection, compared with convenience sampling, allows for reaching participants outside the researcher’s geographic area.

Participants were asked to participate in a study related to fitness tracker information. After reading the consent form, the participants were asked to start the survey. The first question asks participants whether they have owned a fitness tracker. The survey ended for those who indicated they did not own a fitness tracker. The participants were paid USD 0.50 for completing the survey. The participants were randomly assigned to one of two groups (granular or universal). Under the granular privacy scenario, the participants were exposed to a scenario where they could select what fitness information to share and with whom. Next, they were asked how likely they are to share the information under such a scenario. In universal sharing, the participants were told that they could not exclude specific information from sharing. Next, they were asked to indicate their sharing intentions under this scenario. All participants answered the same questions related to independent and control variables. Control variables were sex and prior privacy invasion, included to follow the design of Angst et al [18], within the health care context.

### Data Summary

The final data analysis included 270 valid and complete responses. The participants had to answer questions related to the independent variables, as shown in Figure 1. Next, each participant was exposed to one of two sharing settings. Finally, the participants indicated their intention to share their fitness information with health care providers, depending on the sharing setting to which they were exposed. Qualtrics settings allowed for random assignment, while keeping the number of participants in the two groups similar. In total, 137 participants were assigned to the granular privacy sharing option (select what fitness information to disclose and share and whom to share it with), and 133 were assigned to the universal sharing option (share all personal fitness information with all providers). Of the 270 participants, 77.8% (n=211) were male, and the rest (22.2%, n=59) were female. The majority of the participants were between the age of 25 and 34 years (163 participants), the second-largest group was 35-44 years old (39 participants), 33 participants were between the age of 18 and 24 years, 21 participants were between the age of 45 and 54 years, and 14 participants were 55 years old or older.

### Measurement Model Assessment

SAS software version 9.4 was used to decode the data, and IBM AMOS version 25 was used to run the analysis. Confirmatory factor analyses were used to evaluate the measurement model (Table 1) using all 270 participants. All variables in this study were adapted from prior research (see Table A1 of Multimedia Appendix 1 for measurement items). All latent variables were measured on a 5-point Likert scale. The results of the measurement model showed a good fit [59]. All factor loadings for the latent variables were relatively strong and significant. The comparative fit index (CFI)=.964, root-mean-square error of approximation (RMSEA)=.059, Tucker-Lewis index (TLI)=.953, and χ^2^/df=1.724. These results provided evidence of the validity of the constructs.

**Table 1:** Measurement model.

Latent variable	Item	Loadings	Corrected item–total correlation	Construct reliability	Variable inflation factor
Intention to share (dependent variable)	INT_1	0.82	0.730	0.863	NA^a^
	INT_2	0.782	0.702		
	INT_3	0.867	0.778		
Perceived risk	PR_1	0.804	0.619	0.764	1.047
	PR_2	0.633	0.550		
	PR_3	0.718	0.612		
Perceived benefits	PB_1	0.7	0.616	0.758	1.003
	PB_2	0.69	0.577		
	PB_3	0.753	0.576		
Trust in the system	TR_1	0.733	0.545	0.721	1.029
	TR_2	0.591	0.511		
	TR_3	0.712	0.569		
Prior experience with privacy invasion (control)	PI_1	0.847	0.747	0.850	1.015
	PI_2	0.785	0.699		
	PI_3	0.794	0.710		

^a^NA: not available.

The reliability of constructs was assessed by calculating the composite reliability (CR). The reliability scores for all constructs in the conceptual model exceeded the threshold of 0.7, which indicates strong reliability. The CR scores ranged from 0.721 to 0.863 (see Table 1). In addition, the corrected item–total correlation for each item was calculated based on the construct to which it belonged. All values exceeded the minimum cutoff of 0.5 [60]. Furthermore, the variance inflation factor (VIF) was calculated for each of the independent variables in the measurement model to check for multicollinearity. All VIF values were way below the threshold score of 10. Therefore, there was no evidence for the existence of multicollinearity between variables in this study.

## Results

### SEM Results

**Figure 2:**
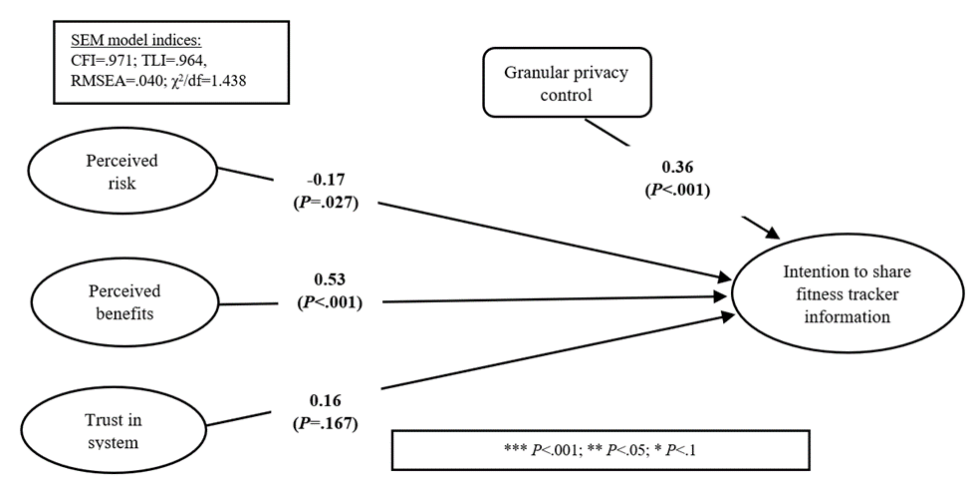
SEM results. CFI: comparative fit index; RMSEA: root-mean-square error of approximation; SEM: structural equation modeling; TLI: Tucker-Lewis index.

Figure 2 shows the results of structural equation modeling (SEM). The model explained 60.4% of the variance (R^2^) in the intention to share.

### Granular Privacy Control

Hypothesis 1 states that increased control results in a higher intention to share. Findings supported this result. The path coefficient for granular privacy control was positive and significant (β_GPC_=.36, *P*<.001), indicating that granular privacy control yields a higher willingness to share personal fitness-related information with health care providers.

### Perceived Risk

Hypothesis 2 proposes a positive relationship between negative perceived risk and the intention to share fitness information. The results provided evidence to support this hypothesis (β_PR_=–.17, *P*=.027), which confirms the impact of the possible risk that involves sharing information via systems and with others.

### Perceived Benefits

Hypothesis 3 argues that perceived benefits will yield a higher intention to share fitness information with health care providers. The estimate for this relationship was positive and significant (β_PB_=.53, *P*<.001), which provides support for the hypothesis. The magnitude of the influence was the highest among all variables, which confirms the importance of benefits for individuals to be willing to share their fitness information.

### Trust in a System

Finally, hypothesis 4 states that trust in the system will have a positive influence on the intention to share personal fitness information with health care providers. However, the results did not provide support for this hypothesis. The estimate was positive but not significant. Thus, trust does not seem to be an issue or a barrier to information sharing in this context. Prior invasion of privacy and sex were used as control variables. It is expected that individuals’ perceptions are influenced by the prior invasion of privacy. Both variables were positively significant.

## Discussion

### Principal Findings

Privacy calculus theory highlights that people weigh benefits and risks when making decisions related to sharing or disclosing personal information. This research finds that both risk and benefits have a significant influence on the intention to share fitness data (see Figure 2). These findings are similar to the findings of other research in the health care and cybersecurity context [18,51]. However, the benefits of sharing are more influential compared to the risks, at least in this sample. This result is positive for health care providers, researchers, and those who need the information to improve population health. User benefits include improved health care quality, more accurate information, more convenience, and better communication. In addition, people who share their fitness information with health care providers may be able to avoid serious problems by allowing the providers to detect problems early. This could result in avoiding increases in health insurance premiums for individuals who share their information.

Cavusoglu et al [21] showed that granular privacy control motivates Facebook users to share more content because they are able to control the content they can share and with whom they can share it. This is in line with the results of this research that show that granular privacy control could motivate people to share their fitness tracker information. Prior research [18,20] has shown that sharing health-related information is perceived by individuals to be risky. The results of this study confirm that sharing fitness information is also perceived to be risky. However, the benefits seem to outweigh the risks.

In addition, this paper integrated CPM with the privacy calculus model. CPM defines the motivation behind individuals’ choice to share or withhold private information. In this paper, participants were randomly assigned to scenarios (granular and universal sharing). Findings showed that granting people greater privacy control acts as a persuasive mechanism to motivate more people to participate in sharing their fitness information. Thus, individuals can engage in behaviors that may improve their well-being, while taking actions to protect their private data. This is an implication for policymakers to enforce granular privacy sharing settings that will allow individuals to participate in such systems and, in turn, observe better health outcomes. In addition, a higher participation rate will allow those applications to be sustainable as they enable more people to benefit from the system.

Trust in the system does not have a significant relationship with fitness information sharing. This finding requires further investigation because it goes against the hypothesis and previous research as it relates to information sharing. However, several explanations are plausible. For example, in this era, most people use apps and systems all the time. Thus, the concept of general trust in the system starts to vanish as systems become part of our daily work and personal routines.

### Limitations and Future Work

This study had several limitations. First, the dependent variable was the intention to share fitness information with health care providers and not actual behavior. However, previous studies have indicated that intention is a strong predictor of actual behavior [61]. Another limitation was that the data were collected online through Amazon MTurk. This could also be associated with selection bias. However, many studies in the health care field have used online data collection methods. In addition, after the COVID-19 situation, online data collection is expected to become more prominent. Furthermore, the integration of fitness apps and systems of health care providers has not been adopted yet, at least not on a large scale. Future work will focus on other aspects of application design and privacy and security settings.

### Conclusion and Contribution

The findings of this study have many implications for practice and the literature. Individuals, generally, choose to share specific information with specific health care providers. Viewed from a privacy perspective, enforcement of granular privacy settings lessens the perceived risk by giving individuals a greater sense of assurance regarding their personal fitness information. This research finds that on average, people are likely to share their fitness information when applications empower them with more control. That is because people naturally prefer to avoid risk. Granular privacy control offers people the ability to mitigate risk. This step will increase their willingness to participate in sharing personal fitness information.

This paper illustrates how providing individuals with granular privacy control can lead to improvement in sharing of fitness information. This could result in improved health outcomes for individuals and the general public. Granular privacy control allows individuals to mitigate the perceived risk involved in the universal sharing of all fitness information.

In general, the perceived risk remains a major barrier to information sharing, even with regard to fitness information. The introduction of granular privacy control could mitigate the negative impact of perceived risk. On the positive side, perceived benefits show the strongest influence on the intention to share fitness information. This indicates that individuals attach sharing fitness information to many benefits. The magnitude of the perceived benefit coefficient is three times stronger than the coefficient of perceived risk. This also has implications for the need for such integration between fitness apps and health care systems. Policymakers may want to consider establishing policies and rules that govern the sharing process.

This research contributes to theory by integrating the privacy model and CPM theory in the context of fitness information sharing. In addition, the study adds to theory by highlighting the impact of granular privacy control on the intention to share fitness information.

## Appendix

Multimedia Appendix 1 Measurement items.

## Abbreviations

CFI: comparative fit index
CPM: communication privacy management
CR: composite reliability
IoT: internet of things
MTurk: Mechanical Turk
RMSEA: root-mean-square error of approximation
SEM: structural equation modeling
TLI: Tucker-Lewis index
VIF: variance inflation factor

## References

[1] Douglas-Walton J A Study of Fitness Trackers and Wearables.

[2] Comstock J (2015). Eight Years of Fitbit News Leading up to Its Planned IPO.

[3] Dormehl L Today in Apple History: It’s Time for Apple Watch.

[4] Vogels E About One-in-Five Americans Use a Smart Watch or Fitness Tracker.

[5] Mccarthy J (2019). One in Five U.S. Adults Use Health Apps, Wearable Trackers.

[6] Asimakopoulos S, Asimakopoulos G, Spillers F (2017). Motivation and user engagement in fitness tracking: heuristics for mobile healthcare wearables. Informatics.

[7] Wu Q, Sum K, Nathan-Roberts D (2016). How fitness trackers facilitate health behavior change.

[8] Naglis M, Bhatiasevi V (2019). Why do people use fitness tracking devices in Thailand? An integrated model approach. Technol Soc.

[9] (2017). Wellness Is The New Luxury: Is Healthy And Happy The Future Of Retail?.

[10] Nermoe K (2018). Millennials: The "Wellness Generation".

[11] Dinh-Le C, Chuang R, Chokshi S, Mann D (2019). Wearable health technology and electronic health record integration: scoping review and future directions. JMIR Mhealth Uhealth.

[12] Gabriele S (2020). Understanding fitness tracker users' security and privacy knowledge, attitudes and behaviours.

[13] Osborne C The Latest Healthcare Data Breaches in 2019/2020.

[14] Dinev T, Hart P (2006). An extended privacy calculus model for e-commerce transactions. Inf Syst Res.

[15] Birelin A (2017). The Benefits of Logging Workouts into a Fitness App.

[16] Perez A (2019). Use a Fitness App to Track Your Workouts? Your Data May Not Be as Protected as You Think.

[17] Hutton L, Price BA, Kelly R, McCormick C, Bandara AK, Hatzakis T, Meadows M, Nuseibeh B (2018). Assessing the privacy of mHealth apps for self-tracking: heuristic evaluation approach. JMIR Mhealth Uhealth.

[18] Angst CM, Agarwal R (2009). Adoption of electronic health records in the presence of privacy concerns: the elaboration likelihood model and individual persuasion. MIS Quarterly.

[19] Abdelhamid M, Kisekka V, Samonas S (2019). Mitigating e-services avoidance: the role of government cybersecurity preparedness. ICS.

[20] Anderson CL, Agarwal R (2011). The digitization of healthcare: boundary risks, emotion, and consumer willingness to disclose personal health information. Inf Syst Res.

[21] Cavusoglu H, Phan TQ, Cavusoglu H, Airoldi EM (2016). Assessing the impact of granular privacy controls on content sharing and disclosure on Facebook. Inf Syst Res.

[22] Li H, Gupta A, Zhang J, Sarathy R (2014). Examining the decision to use standalone personal health record systems as a trust-enabled fair social contract. Decis Support Syst.

[23] Bansal G, Gefen D, Zahedi (2010). The impact of personal dispositions on information sensitivity, privacy concern and trust in disclosing health information online. Decis Support Syst.

[24] Simon SR, Evans JS, Benjamin A, Delano D, Bates DW (2009). Patients' attitudes toward electronic health information exchange: qualitative study. J Med Internet Res.

[25] Orehek J, Charpin D, Gayrard P, Grimaud Ch (1976). Bronchodilator properties of a vasodilator: cetiedil. Nouv Presse Med.

[26] Zulman DM, Nazi KM, Turvey CL, Wagner TH, Woods SS, An LC (2011). Patient interest in sharing personal health record information: a web-based survey. Ann Intern Med.

[27] Dimitropoulos L, Patel V, Scheffler SA, Posnack S (2011). Public attitudes toward health information exchange: perceived benefits and concerns. Am J Manag Care.

[28] Ancker JS, Edwards AM, Miller MC, Kaushal R (2012). Consumer perceptions of electronic health information exchange. Am J Prev Med.

[29] Gerlach J, Widjaja T, Buxmann P (2015). Handle with care: How online social network providers’ privacy policies impact users’ information sharing behavior. J Strateg Inf Syst.

[30] Smith HJ, Dinev T, Xu H (2011). Information privacy research: an interdisciplinary review. MIS Quarterly.

[31] Sun Y, Wang N, Shen X, Zhang JX (2015). Location information disclosure in location-based social network services: privacy calculus, benefit structure, and gender differences. Comput Hum Behav.

[32] Kim D, Park K, Park Y, Ahn J (2019). Willingness to provide personal information: perspective of privacy calculus in IoT services. Comput Hum Behav.

[33] Fox G (2020). “To protect my health or to protect my health privacy?” A mixed‐methods investigation of the privacy paradox. J Assoc Inf Sci Technol.

[34] Abdelhamid M, Gaia J, Sanders GL (2017). Putting the focus back on the patient: how privacy concerns affect personal health information sharing intentions. J Med Internet Res.

[35] Petronio S (2007). Translational research endeavors and the practices of communication privacy management. J Appl Commun Res.

[36] McLeod A, Dolezel D (2018). Cyber-analytics: modeling factors associated with healthcare data breaches. Decis Support Syst.

[37] Dhopeshwarkar RV, Kern LM, O'Donnell HC, Edwards AM, Kaushal R (2012). Health care consumers' preferences around health information exchange. Ann Fam Med.

[38] Angst CM, Block ES, D’Arcy J, Kelley K (2017). When do IT security investments matter? Accounting for the influence of institutional factors in the context of healthcare data breaches. MISQ.

[39] Tversky A, Fox CR (1995). Weighing risk and uncertainty. Psychol Rev.

[40] Caine K, Hanania R (2013). Patients want granular privacy control over health information in electronic medical records. J Am Med Inform Assoc.

[41] Westin Alan (1967). Privacy and freedom. Washington and Lee Law Review.

[42] Laugesen J, Hassanein K (2017). Adoption of personal health records by chronic disease patients: a research model and an empirical study. Comput Hum Behav.

[43] Abdelhamid M (2018). Greater patient health information control to improve the sustainability of health information exchanges. J Biomed Inform.

[44] Slovic P (2000). The Perception of Risk: Risk, Society and Policy.

[45] Brandimarte L, Acquisti A, Loewenstein G (2012). Misplaced confidences. Soc Psychol Personal Sci.

[46] Yi J, Yuan G, Yoo C (2020). The effect of the perceived risk on the adoption of the sharing economy in the tourism industry: the case of Airbnb. Inf Process Manag.

[47] Hansen JM, Saridakis G, Benson V (2018). Risk, trust, and the interaction of perceived ease of use and behavioral control in predicting consumers’ use of social media for transactions. Comput Hum Behav.

[48] Hilverda F, Kuttschreuter M (2018). Online information sharing about risks: the case of organic food. Risk Anal.

[49] Morris John (2016). First Look: Internet Use in 2015.

[50] Lee Y, Queenie Li J (2020). The value of internal communication in enhancing employees’ health information disclosure intentions in the workplace. Public Relat Rev.

[51] Wang J, Huang J, Cheung CSK, Wong WN, Cheung NT, Wong MC (2020). Adoption of an electronic patient record sharing pilot project: cross-sectional survey. J Med Internet Res.

[52] Zhang X, Liu S, Chen X, Wang L, Gao B, Zhu Q (2018). Health information privacy concerns, antecedents, and information disclosure intention in online health communities. Inf Manag.

[53] Morris K, Yamamoto G, Sugiyama O, Luciano S, Tsutsumi T, Ohtsuki R, Kato G, Hiragi S, Okamoto K, Nambu M, Kuroda T (2020). Designing a mobile patient information sharing system using patients community members: perceptions of emergency physicians. Eur J Biomed Inf.

[54] Pavlou PA, Fygenson M (2006). Understanding and predicting electronic commerce adoption: an extension of the theory of planned behavior. MIS Quarterly.

[55] Lin W, Zhang X, Song H, Omori K (2016). Health information seeking in the Web 2.0 age: trust in social media, uncertainty reduction, and self-disclosure. Comput Hum Behav.

[56] Gefen D, Karahanna E, Straub DW (2003). Trust and TAM in online shopping: an integrated model. MIS Quarterly.

[57] Bartek MA, Truitt AR, Widmer-Rodriguez S, Tuia J, Bauer ZA, Comstock BA, Edwards TC, Lawrence SO, Monsell SE, Patrick DL, Jarvik JG, Lavallee DC (2017). The promise and pitfalls of using crowdsourcing in research prioritization for back pain: cross-sectional surveys. J Med Internet Res.

[58] Pew Research Center (2018). Internet/Broadband Fact Sheet.

[59] Hu L, Bentler PM (1999). Cutoff criteria for fit indexes in covariance structure analysis: conventional criteria versus new alternatives. Struct Equ Modeling.

[60] Hair J (2006). Multivariate Data Analysis (Vol. 6).

[61] Rise J, Kovac V, Kraft P, Moan I S (2008). Predicting the intention to quit smoking and quitting behaviour: extending the theory of planned behaviour. Br J Health Psychol.

