# Supplementary material for: Fitness Tracker Information and Privacy Management: Empirical Study
Source: J Med Internet Res. 2021 Nov 16;23(11):e23059. doi: 10.2196/23059 (PMC8663694; doi:10.2196/23059)
Supplement: Multimedia Appendix 1 [file jmir_v23i11e23059_app1.docx]

**Appendix**

Table A1. Measurement Items

| Construct | Code | Items | Adapted |
| --- | --- | --- | --- |
| Intention to share (dependent variable) | Under this setting, as a user……… | | [1] |
|  | INT_1 | I am likely to consent to share my personal fitness information. |  |
|  | INT_2 | I will probably consent to share my personal fitness information. |  |
|  | INT_3 | I am willing to consent to share my personal fitness information. |  |
| Perceived risk | I believe there is a very high risk for sharing personal fitness information to the possibility that.. | | [2] |
|  | PR_1 | Records of personal fitness information could be sold to third parties. |  |
|  | PR_2 | Personal fitness information submitted could be misused. |  |
|  | PR_3 | Personal fitness information could be made available to unknown individuals or companies without your knowledge. |  |
| Perceived benefits | PB_1 | Overall, sharing personal fitness tracker information with healthcare providers will be advantageous. | [3] |
|  | PB_2 | Sharing personal fitness tracker information with healthcare providers will be of beneficial to me. |  |
|  | PB_3 | Sharing personal fitness tracker information with healthcare providers online is a good idea. |  |
| Trust in system | I believe that the system used for managing and exchanging fitness information.... | | [2] |
|  | TR_1 | is trustworthy. |  |
|  | TR_2 | can be relied upon when dealing with my personal health information. |  |
|  | TR_3 | is safe. |  |
| Prior experience with privacy invasion  (control) | When it comes to the privacy invasion of information, **my prior experience**could be characterized as: | | [4] |
|  | PI_1 | definitely victimized. |  |
|  | PI_2 | definitely bad experiences. |  |
|  | PI_3 | definitely invasion of privacy. |  |

**Refrences**

1. Malhotra, N.K., S.S. Kim, and J. Agarwal, *Internet users' information privacy concerns (IUIPC): The construct, the scale, and a causal model.* Information systems research, 2004. **15**(4): p. 336-355.

2. Dinev, T. and P. Hart, *An extended privacy calculus model for e-commerce transactions.* Information Systems Research, 2006. **17**(1): p. 61-80.

3. Abdelhamid, M., *Greater Patient Health Information Control to Improve the Sustainability of Health Information Exchanges.* Journal of biomedical informatics, 2018.

4. Anderson, C.L. and R. Agarwal, *The digitization of healthcare: boundary risks, emotion, and consumer willingness to disclose personal health information.* Information Systems Research, 2011. **22**(3): p. 469-490.
